# Enhancement of Thermal Sealing and Solubility Properties of Konjac-Glucan/Gelatin Films by Hydroxypropyl Cassava Starch Thermoplastic Effect

**DOI:** 10.3390/foods15071254

**Published:** 2026-04-07

**Authors:** Lingxin Yu, Wenxu Gao, Meining Li, Zhiwen Hu, Yang Li, Junhua Li, Jie Pang, Junyan Shi

**Affiliations:** 1 Institute of Agro-Food Science and Technology, Shandong Academy of Agricultural Sciences, Jinan 250100, China; 2 College of Food Science, Fujian Agriculture and Forestry University, Fuzhou 350002, China

**Keywords:** konjac glucomannan, hydroxypropyl tapioca starch, rapid dissolution, heat sealing, food packaging

## Abstract

The burgeoning convenience food sector, particularly in China, has intensified demand for packaging that simultaneously delivers convenience, environmental sustainability, and functional performance. This study addresses this need by developing a novel self-sealing, rapidly soluble food packaging film. The film was prepared using solvent casting technology, with a konjac glucomannan (KGM) matrix as the base material and gelatin (Gel) and hydroxypropyl tapioca starch (HS) as reinforcing agents. Leveraging the thermoplastic effect of HS (its hydroxypropyl side chains disrupt the ordered hydrogen bond network of KGM and Gel, enhancing molecular chain mobility) characterization via FTIR and SEM confirmed successful heat-sealing upon HS incorporation, while dissolution testing validated enhanced dissolution kinetics. The optimal formulation (KGH_3_) exhibited superior mechanical properties (tensile strength (TS): 17.54 MPa) and excellent barrier performance against both light and oxygen transmission compared to pristine KGM and KG control films. Self-sealed pouches fabricated from KGH films preserved edible oil for 65 days, maintaining peroxide values within acceptable limits and demonstrating 48.7% reduction in oxidation compared to KG films. These findings establish KGM–Gel–HS film as promising candidates for adhesive-free, biodegradable packaging of lipid-rich foods.

## 1. Introduction

The expanding global convenience food industry, alongside its rapidly growing market, has intensified consumer demand for packaging that is more convenient, environmentally friendly, and functional [1]. Although traditional convenience food packaging (e.g., polypropylene, polystyrene) provides low production costs and portability, as well as possessing good mechanical/barrier properties, their extended degradation cycle (centuries in natural environments), high fossil resource consumption, and hazardous waste generation (e.g., microplastic pollution, toxic leachates) cause substantial environmental damage [2]. Biopolymer films derived from renewable polysaccharides and proteins offer a compelling alternative, yet their widespread adoption requires overcoming limitations in functionality, mechanical performance, and processing adaptability [3].

Konjac glucomannan (KGM), an economically viable polysaccharide with excellent film-forming capacity and biodegradability, has attracted significant interest [4]. Compared to other polysaccharides (e.g., chitosan, sodium alginate), KGM offers unique advantages: it forms stable films via simple solvent casting without requiring pH adjustment (unlike chitosan) or divalent cation crosslinking (unlike alginate), reducing preparation complexity. Additionally, KGM’s low cost and abundant availability (derived from konjac tubers) make it more industrially scalable than specialty polysaccharides [5]. However, pure KGM films are limited by their functional simplicity, poor mechanical properties, and lack of flexibility. To enhance its performance, KGM is frequently combined with other polysaccharides and proteins (such as chitosan, starch or gelatin (Gel)) to develop multifunctional composite materials through intermolecular interactions [6]. Gel offers good film-forming ability and barrier properties, along with strong water-binding capacity [7]. However, Gel films are often brittle and rigid, resulting in poor extensibility. To improve the extensibility of Gel, it is commonly blended with hydrophilic polysaccharides such as KGM, sodium alginate, chitosan, or sodium carboxymethyl cellulose [8,9]. Li et al. [10] prepared blend films using different proportions of KGM and Gel, and they found that the mechanical properties and heat sealing performance of the blends were the most ideal. Hydroxypropylated tapioca starch (HS) is a chemically modified starch widely used in the food industry, preferred over natural starch or other modified starches (such as carboxymethyl starch) due to its inherent thermoplastic effect—a key feature distinguishing it from unmodified starches. Unlike natural tapioca starch, which forms rigid, tightly packed structures via strong intermolecular hydrogen bonds, HS’s grafted hydroxypropyl groups introduce steric hindrance, disrupting the ordered hydrogen bond network and converting molecular chains from “rigid stacking” to “flexible extension” [11]. This thermoplastic effect not only enhances the flexibility of HS molecules and their compatibility with KGM/Gel matrices [12] but also improves molecular chain mobility—critical for enabling heat-sealing (via interdiffusion of chains at the film interface) and solubility (via loosening of the film’s microstructure). Additionally, the introduction of the hydroxypropyl group introduces additional hydroxyl groups (-OH), significantly enhancing the hydrophilicity of the molecules [13]. These properties make HS an ideal modifier that can simultaneously improve processing performance and functional performance (heat-sealing) and functional performance (solubility) via its thermoplastic regulation of molecular interactions [14].

Recent advances underscore the potential of biopolymer formulations. Nilsuwan et al. [15] demonstrated that incorporating Gel significantly enhances the heat-sealing properties of films. In a separate study, Zhu et al. [16] found that films prepared with hydroxypropyl distarch phosphate (HPDSP) exhibit superior mechanical properties compared to those from native tapioca starch, approaching the performance of traditional polyethylene films. Additionally, HPDSP films showed greater swelling capacity than oxidized or cationic starch, identifying HPDSP as a promising raw material for fully degradable starch-based packaging. Wei et al. [17] discovered that HS, leveraging its molecular structure, lowers heat-sealing temperature, preserves substrate activity, and eliminates the reliance on traditional adhesives in films. However, the specific role of HS’s thermoplastic effect in regulating the structure–property relationships of KGM–Gel–HS ternary films—particularly how it enhances both heat-sealing and solubility—remains inadequately elucidated.

To address these limitations, this study aims to evaluate the impact of HS addition on the self-sealing properties of KGM–Gel films, to develop a novel, self-sealing, and dissolvable food packaging material. The films were characterized using Fourier transform infrared (FTIR) spectroscopy, thermogravimetric analysis (TGA), X-ray diffraction (XRD), and scanning electron microscopy (SEM) to elucidate the influence of HS at the microscopic level. The heat-sealing, dissolution, physical, and barrier properties were measured to investigate the impact of HS addition on the fundamental performance of the films. KGH films with varying HS addition levels were selected to study their heat-sealing parameters and were subsequently applied for edible oil packaging. Compared with petroleum-based packaging materials, the KGM–Gel–HS film developed in this study, which utilizes renewable resources, undergoes functional testing for heat sealing and rapid dissolution. These characteristics are not possessed by the vast majority of petroleum-based packaging materials [2,3]. This work not only provides a theoretical basis for designing KGM-based food packaging films but also offers new insights into regulating film structures through the manipulation of polysaccharide and protein-blending behavior.

## 2. Materials and Methods

### 2.1. Materials

KGM (purity ≥ 90%, Mw = 1.96 × 10^5^ Da, viscosity: 1.0% solution ≥ 32,000 MPa·s) was supplied by Hubei Yizhi Konjac Technology Co., Ltd. (Yichang, China). HS (food grade, Mw = 6.66 × 10^2^ Da, viscosity: 1.0% solution ≥ 2300 MPa·s, degree of substitution (DS) = 0.20 ± 0.02, in line with the national standard GB 29937-2013 [18]) was purchased from Nantong Gaofeng Biotechnology Co., Ltd. (Nantong, China). Gel (chemical grade, Mw = 5.63 × 10^4^ Da, viscosity: 1.0% solution ≥ 8 MPa·s) was provided by Shanghai Sinopharm Chemical Reagent Co., Ltd. (Shanghai, China). Glycerol (analytical grade) was obtained from Shanghai Sinopharm Chemical Reagent Co., Ltd. (Shanghai, China).

### 2.2. Film Solution Preparation

To prepare the KGM base solution, 0.6 g of KGM powder was first dispersed in distilled water. The mixture was transferred to a thermostatic magnetic stirring vessel, where it was stirred at 40 °C and 300 rpm for 1 h to ensure complete dissolution. Following this, 1% (*w*/*v*) glycerol (serving as a plasticizer) was added to the KGM solution, and stirring was continued for an additional 30 min; this yielded a 0.6% (*w*/*v*) KGM stock solution.

We sought to ensure good compatibility between KGM and Gel, as well as the stable film-forming ability of the binary matrix. These were determined through preliminary screening experiments. The weight ratio of KGM to Gel was set at 3:1. The Gel was first dissolved in 100 mL of the 0.6% (*w*/*v*) KGM stock solution, and the mixture was transferred to a 40 °C water bath. A low-speed electronic stirrer (300 rpm) was used to agitate the mixture for 1 h, allowing sufficient hydration of both components to form a homogeneous KG mixture.

Based on the effect between the film-forming components and the optimization of the target performance in the research, the HS matrix was introduced to prepare the KGM–Gel–HS (KGH) solution. The homogeneous KG mixture was first cooled to room temperature. HS was then added to the KG mixture at three different HS-to-Gel mass ratios: 8:2 (denoted as KGH_1_), 7:3 (KGH_2_), and 6:4 (KGH_3_). Each mixture was stirred for 30 min to disperse the HS uniformly. Subsequently, the mixture was transferred to a thermostatic magnetic stirring vessel, where it was heated to 80 °C and stirred at 300 rpm for 1 h; this step ensured full hydration and interaction between all components, resulting in a stable KGH solution.

### 2.3. Preparation of Thin Films

We then spread 60 mL of the thin film solution evenly onto the plate and dried it in an electric thermostatic blast drying oven (Model DFG-9076A, Shanghai Jinghong Laboratory Equipment Co., Ltd., Shanghai, China) at 50 ± 5 °C for 12 h. After drying is complete, the film is peeled off and placed in a desiccator containing a solution of sodium bromide (NaBr) at the bottom. It is left to stand at 25 °C and a relative humidity of 50 ± 2% for 24 h to reach equilibrium. Then, it is used for subsequent experimental operations.

### 2.4. Microstructural Characterization

FTIR spectroscopy (Nicolet iS20, Thermo Fisher Scientific, Waltham, MA, USA) analyzed functional group interactions in films, with a scanning range of 400–4000 cm^−1^ and resolution of 4 cm^−1^. Thermal stability was evaluated via TGA (TG 209 F3, Netzsch, Selb, Freistaat Bayern, Germany) under nitrogen (20 mL/min) from 20 to 600 °C at 10 °C/min. XRD (Rigaku Smart Lab SE, Osaka, Japan) characterized microcrystalline structures using a Cu target, scanning at 2°/min over 10–80°. SEM (ZEISS Sigma 300, Oberkochen, Germany) observed surface, cross-section, and heat-sealed cross-section microstructures. Samples were gold-sputtered and observed at 3 kV.

### 2.5. Heat-Sealing and Dissolution Properties

#### 2.5.1. Heat Seal Strength

The test method was adapted from Liu et al. [19]. Film samples were trimmed into two 10 mm × 50 mm strips, stacked with aligned edges, and heat-sealed near the edge using a heat-sealing machine (FR-510, Zhongmin Machinery Co., Ruian, China) at 155 °C for 4.0 s with a seal width of 10 mm under a pressure of 300 kPa.

Heat seal strength was determined using a peel test adapted from Lu et al. [20] with modifications. Samples were clamped in a universal testing machine (WDW-5, Changchun Xinte Testing Machine Co., Ltd., Changchun, China) with the two ends separated at 180°. Testing was conducted with an initial grip separation of 80 mm and a crosshead speed of 10 mm/min. Tests were performed in quintuplicate, and results were averaged. The maximum force required to cause seal failure was defined as heat seal strength, expressed in N/m and calculated using Equation (1):

(1) $$\mathrm{Heatseal}\;\mathrm{strength}\;=\;\frac{\mathrm{PF}}{W}$$

where PF refers to the peak force (the maximum force recorded when the heat-sealed sample fractured during the tensile test, unit: N), and W denotes the width of the heat-sealed area of the sample (unit: mm).

#### 2.5.2. Dissolution Test

The dissolution test was conducted following the method of Natsia et al. [21]. A 10 cm × 10 cm film sample was placed in one of three simulated dissolution media maintained at 60 °C: water, 30% ethanol (*v*/*v*), or 50% ethanol (*v*/*v*). Under magnetic stirring (100 r/min), the time required for the film to completely disperse and dissolve in the medium, with no visible solid particles remaining, and to form a uniform solution/suspension is recorded as the dissolution time (unit: s).

### 2.6. Thermophysical Properties

#### 2.6.1. Thickness and Uniformity

Film thickness was measured using a digital micrometer (Mitutoyo, 331-251, Kawasaki, Kanagawa, Japan). Five symmetrical points (including the center) were measured on each film, with thickness expressed in micrometers (μm). The average value was recorded as film thickness, and the standard deviation represented uniformity.

#### 2.6.2. Mechanical Properties

Tensile strength (TS) and elongation at break (EAB) were measured in accordance with GB/T 1040.3—2006 [22] “Determination of tensile properties of plastics—Test conditions for films and sheets”. The films were cut into 10 mm × 100 mm strips as test samples, and the measurements were conducted using a universal testing machine at a crosshead speed of 20 mm/min. Five replicates were tested per sample, and average values were calculated. TS was calculated using Equation (2):

(2) $$\mathrm{TS}\;=\;\frac{F_{\max}}{W\times t}$$

The unit of TS here is MPa, where F_max_ represents the maximum load at sample fracture (N), W is the sample width (mm), and t denotes the sample thickness (mm).

EAB was calculated using Equation (3):

(3) $$\mathrm{EAB}\;=\;\frac{{L_{1}-L}_{0}}{L_{0}}\times 100\%$$

The unit of EAB here is %, where L_0_ represents the initial length of the sample (mm), and L_1_ denotes the length at the point of fracture (mm).

#### 2.6.3. Contact Angle

The contact angle was determined according to the method of Lu et al. [23] and modified accordingly. Film samples were trimmed into 20 mm × 20 mm squares and analyzed using a JY-80 contact angle goniometer (SINDIN, SDC-350KS, Jingtuo, Tianjing, China). The water contact angle (WCA) and oil contact angle (OCA) were determined using the hanging drop method. WVC involves dripping 6 microliters of distilled water onto the film surface and then taking images immediately (0 s) and after a 45 s equilibration period. OCA involves dripping 4 microliters of soybean oil onto the film surface and then taking images immediately (0 s) and after a 45 s equilibrium period. For each sample, measurements were taken at two distinct locations with 3–5 replicates per location.

### 2.7. Barrier Properties

#### 2.7.1. Light Barrier Efficiency

Film samples were trimmed into 10 mm × 20 mm rectangles and mounted flat against the inner wall of a quartz cuvette. Transmittance spectra of the films were first recorded using a UV–visible spectrophotometer over a scanning range of 200–750 nm (UV region: 200–400 nm; visible region: 400–750 nm). A blank quartz cuvette was used as the reference (transmittance = 100%). Each sample was measured in triplicate, and the average transmittance value was calculated. Light Barrier Efficiency was calculated using Equation (4):

Light Barrier Efficiency = (1 − Transmittance) × 100% (4)

The unit of Light Barrier Efficiency here is %. Transmittance represents the light transmittance.

#### 2.7.2. Water Vapor Permeability

The Water Vapor permeability (WVP) of the biopolymer films was evaluated using the gravimetric method, following the ASTM E96 standardized desiccant approach, with modifications based on Deng et al. [24]. At 25 °C, anhydrous calcium chloride (5.0 g) was placed into a weighing bottle, which was sealed with the test film and weighed. The sealed bottle was placed in a desiccator containing ultra-pure water and equilibrated for 24 h before being weighed again. WVP was calculated using Equation (5):

(5) $$\mathrm{WVP}\;=\;\frac{∆m\times x}{A\times t\times ∆p}$$

where WVP is the Water Vapor permeability (g·mm/m^2^·24 h·kPa), Δm denotes the mass change in the weighing bottle before and after testing (g), x represents the thickness of the film sample (mm), A is the test area of the film sample (m^2^), t is the equilibration time (h), and ΔP is the water vapor pressure difference across the film at 25 °C, approximately 3.168 kPa.

#### 2.7.3. Oxygen Permeability Rate

Oxygen permeability (OP) was determined according to the method of Tang et al. [25] with modifications. An oxygen absorbent mixture composed of iron powder, activated carbon, and sodium chloride at a mass ratio of 1:2:3 (total mass 6 g) was placed into a weighing bottle, which was then sealed with the test film. The bottle was stored in a desiccator containing a saturated barium chloride solution to maintain constant humidity for 24 h. The mass change was recorded, and OP was calculated using Equation (6):

(6) $$\mathrm{OP}\;=\;\frac{{∆m}_{0}}{A\times t}$$

where OP represents the Oxygen permeability (g/m^2^·24 h), Δm_0_ denotes the mass change in the weighing bottle before and after the test (g), t is the test duration (24 h), and A is the bottle mouth area (m^2^).

### 2.8. Antioxidant Property

DPPH radical scavenging activity was measured according to the method of Wang et al. [26] with slight modifications. A film sample (50 mg) was dissolved in 10 mL of 0.1 mmol/L DPPH–ethanol solution and reacted in the dark for 30 min. The absorbance of the reaction mixture was measured at 517 nm using a UV–visible spectrophotometer. Radical scavenging activity (RSA) was calculated using Equation (7):

(7) $$\mathrm{DPPH}\;\mathrm{radical}\;\mathrm{scavenging}\;\mathrm{activity}\;(\%)\;=\;\frac{A_{0}-A_{i}}{A_{0}}\times 100\%$$

where A_0_ represents the absorbance of the blank control and Aᵢ denotes the sample absorbance.

### 2.9. Seasoning Oil Packaging Application

#### 2.9.1. Film Packaging for Flavored Oils

Following the method of Wang et al. [27]. A 12 cm × 12 cm square film was folded in half and heat-sealed 10 mm from the remaining edges using a heat-sealing machine at 0.30 MPa and 155 °C for 4.0 s to produce a pouch containing 5 mL of edible oil.

#### 2.9.2. Determination of Peroxide Value

The peroxide value (POV) of oils was determined using the experimental method developed by Zhang et al. [28]. Oil-filled pouches were stored at 25 °C in the dark for 65 days. After storage, the edible oil was retrieved, and its POV was determined according to the method of Nowzari et al. using Equation (8). Results were expressed as meq peroxide/1000 g lipid.

(8) $$\mathrm{POV}=\frac{V\times N\times 1000}{W}$$

where POV is the peroxide value (g/100 g), V represents thiosulfate for titration, N denotes normality of thiosulfate, and W is weight (g) of lipid.

#### 2.9.3. Determination of Soluble Solids in Flavored Oil Packages

The dissolution performance of the film flavored oil bag was evaluated by measuring the soluble solid content (SSC), following the refractive method described in GB/T 12143.1-2008 [29] and with appropriate optimization. The packaged film flavored oil bag samples were placed in 50 mL of distilled water (100 °C) and subjected to magnetic stirring (100 r/min) until the film completely dissolved. The solution was placed in a 60 °C water bath for ultrasonic extraction for 20 min, and the mixed solution was centrifuged for 10 min (4000 r/min, 10 min) to separate the oil phase and filter the supernatant with a 0.45 micron filter membrane. The SSC value of the filtrate was measured using a calibrated digital refractometer at 20 °C and expressed in °Brix.

### 2.10. Statistical Analyses

All experiments were performed at least in triplicate. Data are presented as mean ± standard deviation. Graphs were generated using Origin 2021 software. Statistical analysis was conducted with SPSS 26.0 software using one-way analysis of variance (ANOVA) followed by Duncan’s multiple range test, with significance defined at *p* < 0.05.

## 3. Results and Discussion

### 3.1. Microstructural Characterization of Films

**Figure 1:**
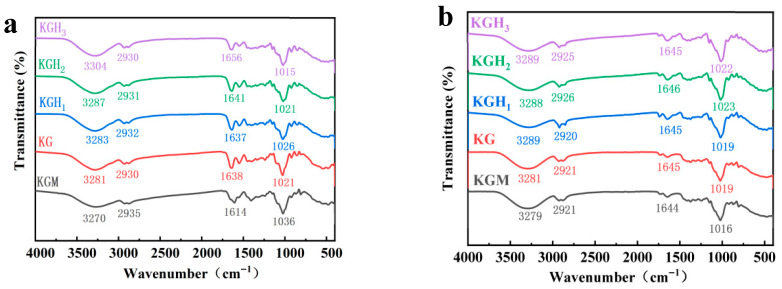
(**a**) FTIR spectra of different films; (**b**) FTIR spectra of different films after heat sealing.

The physicochemical properties of films are governed by the intermolecular interactions of their film-forming components. Figure 1a presents the FTIR spectra of the films. The pure KGM film exhibited a broad peak at 3270 cm^−1^, corresponding to the stretching vibration of numerous -OH groups in KGM molecules. This broad, intense peak indicated dense intermolecular hydrogen bonding in KGM [30]. After Gel addition, the peak near 3300 cm^−1^ intensified, and the -OH stretching vibration band shifted to a higher wavenumber. With increasing HS content, the peak at 3270 cm^−1^ shifted to 3304 cm^−1^, likely because the abundant -OH groups in HS introduced new hydrogen-bonding interactions with -OH groups in KGM and Gel molecules, altering the vibrational environment of -OH [31]. The peak at 2935 cm^−1^ shifted to 2930 cm^−1^, possibly attributed to alkyl chains in HS. Variations in HS content may have affected the number and arrangement of alkyl chains in the films, thereby influencing C-H bond vibrational absorption. The bands near 1642 cm^−1^ and 1022 cm^−1^ corresponded to C-O and C-C-O stretching vibrations in the films. The amide I band (1600–1700 cm^−1^) is characteristic of Gel [32]. With increasing HS content, the band at 1637 cm^−1^ shifted to 1656 cm^−1^. This shift was primarily due to interactions between amide groups in Gel molecular chains and HS, which altered the C=O bond force constant and thus shifted the vibrational peak position. Characteristic vibrational peaks appeared in the 1000–1200 cm^−1^ range. HS introduction disrupted the ordered arrangement of glycosidic bonds in KGM, causing a redshift and decreased intensity of the glycosidic bond vibrational peak. This indicated destruction of the KGM crystalline region and increased molecular disorder [33]. The difference in interaction between the film-forming substances will affect the heat sealing performance of the film, which will be discussed below.

The FTIR spectra of KGM-based films with different HS mass ratios after heat sealing are shown in Figure 1b. After heat sealing, the pure KGM film exhibited a broad peak at 3279 cm^−1^, corresponding to -OH stretching vibrations. This broad, intense peak indicated dense intermolecular hydrogen bonding in KGM. After heat sealing, the film with added Gel exhibited a peak shift to 3289 cm^−1^, and the peak shape was slightly narrower. This might be because the -NH_2_ groups of Gel formed hydrogen bonds with the -OH groups of KGM, disrupting the original hydrogen bond network structure of the KGM film and thus increasing the wavenumber of the -OH vibrational peak while narrowing the peak shape [34]. With the gradual increase in HS content, the peak position stabilized at 3288–3289 cm^−1^, and there was no significant fluctuation in peak intensity. This indicated that after heat sealing, the interactions between the -O-CH_2_-CH(OH)-CH_3_ groups of HS and the hydrogen bonds of KGM and Gel had become stable, and the consistency of functional group vibration was improved [35].

Comparison of FTIR spectra before and after heat sealing revealed that -OH vibrational peak (3200–3500 cm^−1^) and -CH_2_ vibrational peak (2900–3000 cm^−1^) positions became more concentrated (e.g., the peak positions of the KGH series films were close to 3288–3289 cm^−1^ and 2920–2926 cm^−1^). This phenomenon indicated that heat sealing promoted the tight entanglement of HS, Gel, and KGM molecules [36], improving functional group vibrational consistency and enhancing overall peak intensity. This reflected shortened intermolecular distances under heat-sealing pressure, with hydrogen-bonding and van der Waals interactions becoming more significant. After heat sealing, the Gel amide I band (1600–1700 cm^−1^) and KGM glycosidic bond peak (1019–1023 cm^−1^) positions remained essentially stable. This indicated that the 155 °C sealing temperature did not cause the denaturation of Gel protein or the breakage of KGM glycosidic bonds, and the heat sealing process of the films exhibited structural stability.

**Figure 2:**
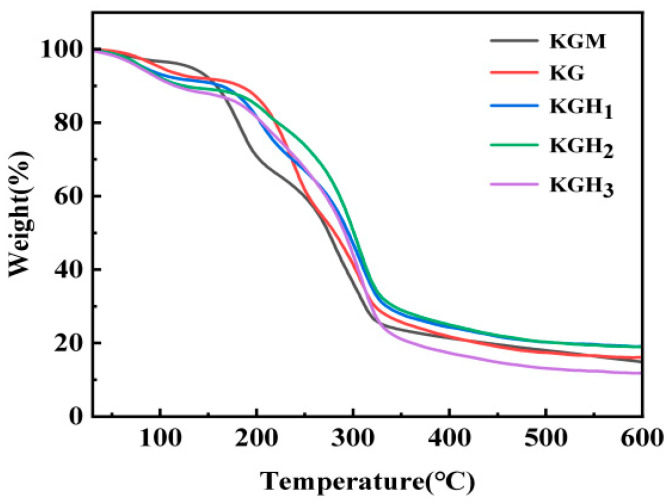
TGA curves of different films.

Figure 2 presents the TGA curves of the films. The KGM film exhibited a typical degradation characteristic of polysaccharide main chains in the temperature range of 200–350 °C [37], with a weight loss rate of 60%. The addition of gelatin resulted in a thermal decomposition temperature range overlapping with that of KGM. Compared to KGM films, the mass loss rate decreased while the final residual mass increased, consistent with FTIR (Figure 1a) results, indicating stabilizing effects. This might be due to the fact that the width of the -OH stretching vibration peak in the KG film (reducing from approximately 3270 cm^−1^ to approximately 3300 cm^−1^) indicates the formation of a denser intermolecular hydrogen bond network. This network, by restricting the mobility of the molecular chains during the thermal degradation process, enhances the thermal stability of the film, thereby reducing the mass loss rate and increasing the residual mass [34]. Notably, the residue content of KGM and KG films is very similar. This is because the two effects of Gel addition offset each other in terms of thermal stability. Gel initially disrupts part of KGM’s original hydrogen bond network (weakening stability), but simultaneously forms new KGM–Gel hydrogen bonds (strengthening stability), resulting in comparable residual mass between the two films. After HS introduction, thermal stability initially increased then decreased, while residual mass gradually decreased. Among the samples, KGH_1_ exhibited the slowest mass loss rate at medium–high temperatures and highest final residual mass (20%), whereas KGH_3_ showed poorest thermal stability, fastest mass loss rate at medium–high temperatures, and lowest final residual mass (10%). This might be because the chemical chains of HS with high content rupture and carbonize rapidly at high temperatures [17], and excessive HS disrupted the original hydrogen bond network of KGM–Gel, leading to a decrease in the overall thermal stability.

**Figure 3:**
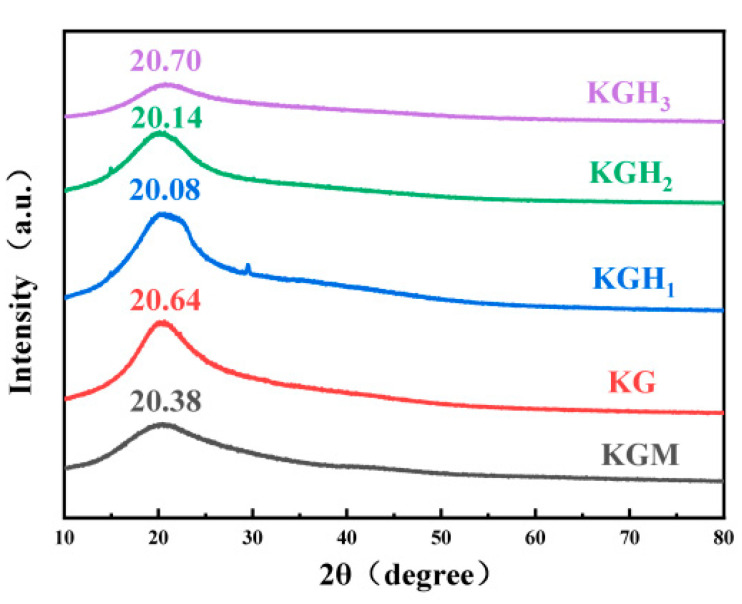
XRD patterns of different films.

XRD was employed to examine the crystalline domains of molecular chains introduced by HS [38]. Figure 3 shows the XRD patterns of the films. All films exhibited similar diffraction characteristics. The KGM film displayed a characteristic diffraction peak at 2θ = 20.38°, attributed to its semicrystalline nature [39]. After the addition of Gel, the characteristic diffraction peak appeared at 2θ = 20.64°, shifting to a higher 2θ value compared to the peak of KGM. Gel disrupted KGM’s original hydrogen bond network and formed new cross-links, tightening molecular chain packing and increasing relative crystallinity. After HS introduction, the characteristic diffraction peak shifted from 2θ = 20.64° to 2θ = 20.08°. HS’s -O-CH_2_-CH(OH)-CH_3_ side chains inserted into the KG network, weakening inter-chain interactions and loosening crystalline domain packing. The low-content HS -O-CH_2_-CH(OH)-CH_3_ groups disrupted the original molecular order. The crystallinity of the film decreases and the molecular arrangement becomes disordered, which is consistent with the broadening of the -OH stretching vibration peak (approximately at 3287 cm^−1^) in the FTIR (Figure 1a). With the increase in HS content, the peak intensity first increased and then decreased: the maximum peak intensity was observed in KGH_1_ (2θ = 20.08°), while the peak intensity of KGH_3_ (2θ = 20.70°) was lower than that of KGH_1_. This is because low-content HS (-O-CH_2_-CH(OH)-CH_3_ groups) moderately disrupts the original molecular order of KG films but promotes local chain packing (increasing peak intensity), whereas high-content HS side chains form new hydrogen bonds but introduce steric hindrance, disrupting the continuity of crystalline domains—thus, relative crystallinity recovers to the KG film level but remains lower than KGH_1_. This demonstrates that HS’s flexible side chains facilitate local chain packing under low-concentration conditions, while high concentration may induce steric hindrance [40].

**Figure 4:**
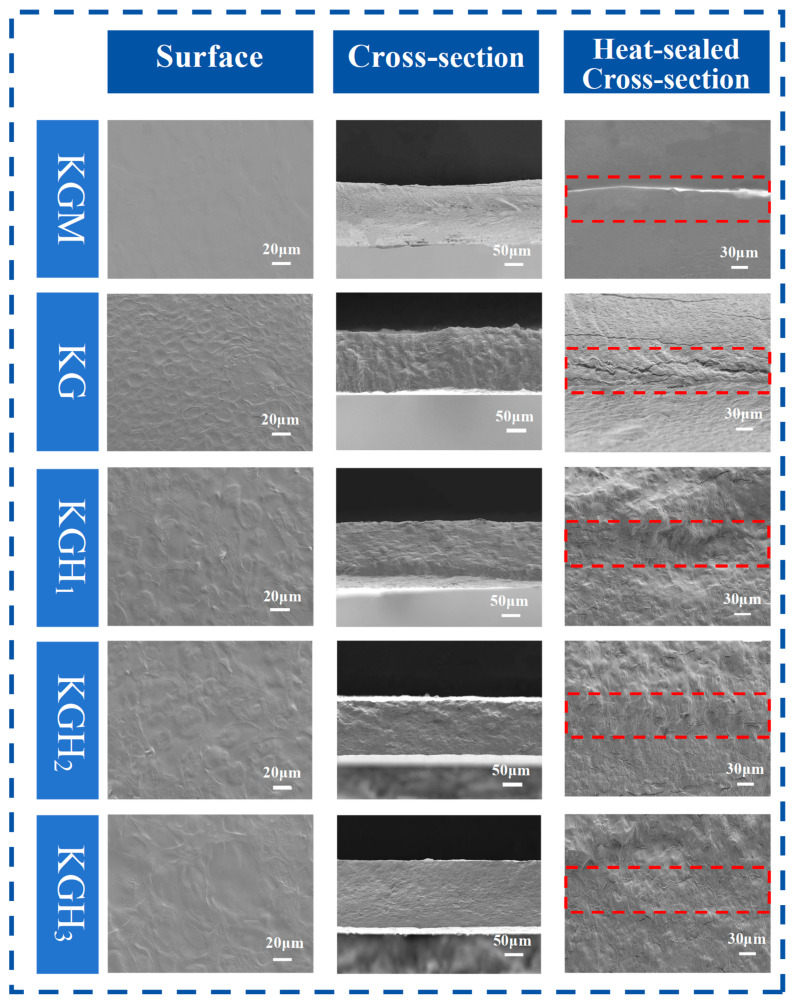
The SEM images of the surface, cross-section and the cross-section after heat sealing of the film (the red box part represents the heat-sealed area).

SEM observation (Figure 4) revealed changes in film microstructure. KGM film shows a relatively loose, non-uniform texture; after the addition of Gel, the surface of the KG film exhibits a more compact and homogeneous texture. With increasing HS addition, film surfaces became more compact and uniform. KGH_3_ surface exhibited particularly high orderliness and uniformity. This might be because the increased content of HS filled some gaps, and the Gel of HS enhanced the compatibility of various components. Cross-sectional morphology revealed that with increasing HS content, films transitioned from porous, irregular layered structures to compact cross-sectional structures. This indicated that HS addition effectively promoted improved bonding between HS and other components as well as between HS molecules. Consistent with the conclusion obtained from FTIR (Figure 1a), HS in the films enhanced the compatibility of the films to a certain extent.

Adhesive-free heat sealing achieves sealing by heating to promote mutual diffusion and entanglement of film molecular chains. SEM cross-sectional images directly reflect heat-sealed interface bonding state (Figure 4). The surfaces of KGM and KG films were relatively rough; after the introduction of HS, the surfaces of the films became smoother and flatter, providing a larger heat-sealing contact area. The pure KGM film exhibited an obvious double-layer structure, and the heat-sealed interface of KG was loose and discontinuous. With the increase in HS content, the gaps in the heat-sealed cross-section narrowed, presenting a denser and more fused structure. Among them, the heat-sealed interface of KGH_3_ showed the optimal uniform and continuous state. This might be because the intermolecular forces between KGM and Gel drove partial heat sealing, but the diffusion ability of molecular chains was limited during heat sealing, resulting in a loose and discontinuous cross-section. HS thermoplasticity provided additional plasticizing sites for molecular chains [41]; as HS content increased, molecular chain entanglement and interface bonding enhanced, strengthening intermolecular bridging [42]. The heat-sealed interface structure was thus regulated through dual plasticization and molecular bridging effects.

### 3.2. Heat-Sealing and Dissolution Properties of Films

**Figure 5:**
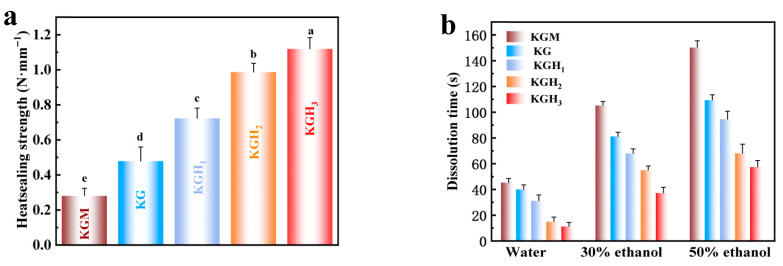
(**a**) Heat seal strength of different films; (**b**) dissolution rate of different films in 60 °C water, 30% ethanol (*v*/*v*), and 50% ethanol (*v*/*v*). Different lowercase letters (a–e) indicate significant differences (*p* < 0.05) among groups.

Adhesive-free heat sealing is a critical indicator of packaging film practicality. Heat seal strength was evaluated by peel testing, with results shown in Figure 5a. Compared with KGM, Gel introduction in KG increased heat seal strength by 58%. This might be because the molecular chains of Gel contained flexible segments, which could undergo thermal diffusion and entanglement with KGM molecular chains during heat sealing [43]. Simultaneously, Gel polar groups (-NH_2_, -COOH) formed hydrogen bonds with KGM -OH groups, enhancing heat-sealed interface bonding force. With increasing HS content, heat seal strength continued to rise, reaching 1.12 N/mm in KGH_3_. In the FTIR results of the sealed film (Figure 1b), the high HS content promoted the reformation of uniform intermolecular hydrogen bonds (the KGH_3_-OH stretching peak became narrower), enhancing the interfacial adhesion between the film layers during the sealing process. In the SEM results of the sealed film (Figure 4), adding HS made the film surface more compact (compared with KGM), thereby reducing surface defects and increasing the contact area between the sealing layers. The results of the FTIR and SEM of the sealed film (Figure 1b and Figure 4) indicate that HS helps improve the sealing performance of the film. This substantial enhancement stems from HS’s plasticization-promoted diffusion [44,45]: the hydroxypropyl groups increased chain flexibility and thermal motion, enabling deeper interpenetration of KGM and Gel chains during sealing. Simultaneously, the increased contact area from HS-induced thickness enhancement contributed to mechanical robustness.

Dissolution performance is crucial for edible packaging applications. Three simulated media—water at 60 °C, 30% ethanol (*v*/*v*), and 50% ethanol (*v*/*v*)—were used to investigate dissolution characteristics of films with different HS ratios. The trends of film dissolution over time in different media are shown in Figure 5b. Data revealed that medium polarity and solvating power directly affected dissolution rate; all films disintegrated faster in 60 °C water than in ethanol solutions. This occurred because water’s stronger polarity enabled rapid disruption of intermolecular hydrogen bonds and electrostatic interactions, causing film structure swelling and dissolution. In contrast, the steric hindrance of ethanol molecules hindered the dissolution and diffusion of film components, leading to a significant decrease in the dissolution rate. KGM film exhibited longer dissolution times (40 s in 60 °C water; 110 s in 50% ethanol), while KG film showed even longer times (46 s; 150 s). This might be attributed to the hydrophilicity of KGM [46]; the addition of Gel made its structure with KGM more compact (Figure 3 and Figure 4), thereby slowing down the dissolution rate. HS addition greatly shortened KGH film dissolution times (KGH_3_: 12 s in 60 °C water; 57 s in 50% ethanol). Although HS addition increased molecular ordering (Figure 3), introduced hydrophilic groups (-O-CH_2_-CHOH-CH_3_) facilitated solvent penetration, weakened intermolecular forces, and reduced thermal stability (Figure 2)—all promoting film dissolution and dissolution.

### 3.3. Characterization of Thermophysical Properties of Films

Good mechanical properties are critical for packaging applications [47]. Table 1 shows TS values and EAB of different films. KGM film TS was 5.60 ± 1.20 MPa and KGH film TS increased from 9.40 ± 2.04 to a maximum of 18.21 ± 1.00 MPa, then decreased to 17.54 ± 1.48 MPa. KGM film EAB of 53 ± 4.77%, KG film EAB of 47.30 ± 5.41%, and KGH_1_, KGH_2_, and KGH_3_ films EAB of 32.95 ± 3.55%, 34.81 ± 3.01%, and 30.00 ± 5.81%, respectively. Data indicate that KGM films had lower strength but better flexibility than KGH films. Initial KGH film TS increase may be attributed to ternary hydrogen bond network formation among HS -O-CH_2_-CH(OH)-CH_3_ side chains, KGM -COCH_3_ groups, and Gel -NH_2_/-COOH groups. Subsequent TS decrease may be due to steric hindrance between excessive HS -O-CH_2_-CH(OH)-CH_3_ side chains and KGM mannose units (Figure 1a). Lower KG film EAB compared with KGM may result from KGM–Gel molecular chain entanglement, which impedes chain sliding and rearrangement under external force, reducing ductility [48]. Lower KGH_1_–KGH_3_ film EAB values compared with KGM and KG may be because HS-introduced -O-CH_2_-CH(OH)-CH_3_ groups competitively formed hydrogen bonds with KGM and Gel, diluting original hydrogen bonds and decreasing network continuity. Notably, compared with biobased materials in Table 1, KGH_2_’s TS (18.21 MPa) surpasses PHA (14.3 MPa) and PBSA (13 MPa), while its EAB (34.81%) is far higher than brittle PLA (8%); versus typical petroleum-based plastics (e.g., LDPE: TS 7–15 MPa, EAB 200–500%), KGH_2_ matches their strength but offers unique biodegradability, balancing performance, and sustainability.

**Figure 6:**
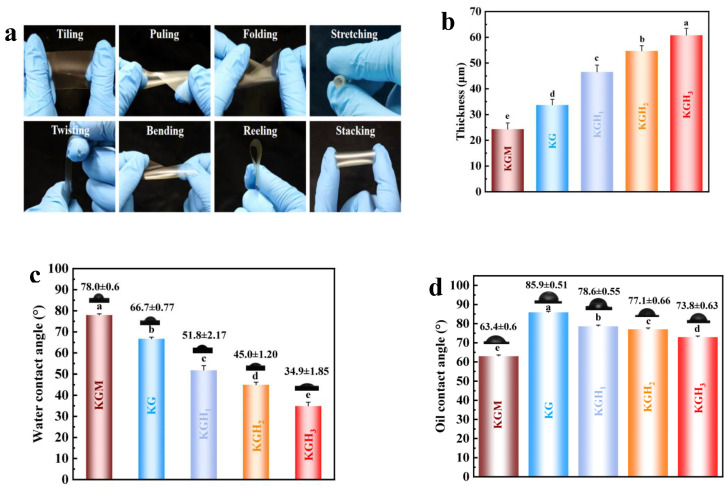
(**a**) Various deformations of KGH films; (**b**) different film thicknesses; (**c**) WCA of different films; (**d**) OCA of different films. Different lowercase letters (a–e) indicate significant differences (*p* < 0.05) among groups.

Thicknesses of KGM, KG, KGH_1_, KGH_2_, and KGH_3_ films are shown in Figure 6b. Film thickness scaled proportionally with HS content, while maintaining uniform morphology (standard deviations < 5%).

In general, higher WCA indicates greater surface hydrophobicity [49]. The WCA of the films is shown in Figure 6c. Since all WCA were below 90°, the films were hydrophilic [50]. Among the five films, KGM film had the highest WCA (78°), while KG film WCA decreased by 14.5% with Gel introduction. The wetting of hydrophilic surfaces increases with the increase in roughness [51]. Gel addition increased dispersed particles, creating a rougher surface (Figure 3 and Figure 4) and reducing WCA. For KGH_1_–KGH_3_, WCA decreased gradually with HS addition. This might be because HS had high affinity for water, resulting in higher wettability [52]. This conclusion was consistent with the result of the dissolution experiment (Figure 5b). The WCA of KGH_2_ (45.0 ± 1.20°) and KGH_3_ (34.9 ± 1.85°) showed a statistically significant difference (*p* = 0.02 < 0.05).

Figure 6d shows OCA of the membranes with different formulations. OCA of the pure KGM membrane is 63.4°. After the introduction of Gel, the contact angle increases to 85.9°. This might be because the hydrogen bond between Gel and KGM temporarily enhances the oil repellency of the surface. As the content of HS increases, the contact angles of KGH_1_, KGH_2_, and KGH_3_ are successively 78.6°, 77.1°, and 73.8°, with KGH_1_ having the highest contact angle. The protein molecules of Gel contain a large amount of -NH_2_ and carboxyl groups -COOH. After forming strong hydrogen bonds with the -OH of KGM, they partially cover the non-polar fragments of KGM, while the polar groups of the protein enhance the polar interfacial energy of the membrane surface. Therefore, the contact angle of the KG membrane increases. The -O-CH_2_-CH (OH)-CH_3_ of HS contains non-polar -CH_2_-CH_3_. As the content of HS increases, -CH_2_-CH_3_ gradually exposes on the membrane surface, reducing the polar interfacial energy of the membrane surface. Therefore, the contact angles of KGH_1_ to KGH_3_ continue to decrease, with KGH_1_ having the most -CH_2_-CH_3_ exposed and the contact angle reaching 73.8°.

**Table 1:** Comparison of TS, EAB and mechanical properties of different films with commercial films.

Film Type	Tensile Strength (MPa)	Elongation at Break (%)	Reference
KGM	5.60 ± 1.20	53.00 ± 4.77	-
KG	9.40 ± 2.04	47.30 ± 5.41	-
KGH_1_	14.64 ± 1.60	32.90 ± 3.55	-
KGH_2_	18.21 ± 1.00	34.80 ± 3.01	-
KGH_3_	17.54 ± 1.48	30.00 ± 5.81	-
PLA	60.7	8.0	[53]
PHA	14.3	298.0	[54]
PBSA	13.0	700.0	[55]
LDPE	7.6	83.0	[56]

### 3.4. Barrier Properties of Films

**Figure 7:**
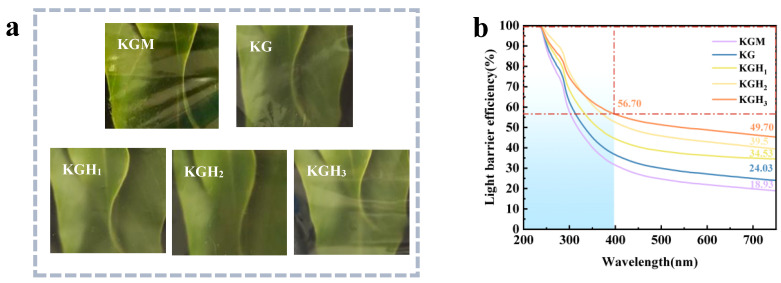
(**a**) Optical diagram of different films (the films were attached to a fresh green leaf to intuitively display their transparency and appearance); (**b**) light barrier efficiency of different films. The red dashed lines in (**b**) indicate the light barrier efficiency of 56.70% at a wavelength of 400 nm, and the light blue shaded area represents the UV-Vis region (200–400 nm).

For the packaging of oil-based foods, the light-blocking property has a significant impact on the preservation of food [57]. Figure 7b shows that the KGM and KGH films have similar light-blocking properties within the wavelength range of 200–750 nanometers. The curve shows that the light-blocking rate in the ultraviolet light region of 200–400 nm is much higher than that in the visible light region of 400–750 nm. The light-blocking rate of all films near 200 nm is close to 100%, indicating that the films have extremely strong barrier capabilities against short-wave ultraviolet light. As the wavelength increases, the light-blocking rate gradually decreases, but KGH_3_ still maintains a high light-blocking rate of 56.70% at 400 nm. With the increase in HS addition amount, the light-blocking rate in the full wavelength range shows a significant upward trend. The light-blocking rate of the pure KGM film is the lowest (only 18.93% at 700 nm), and its ability to block visible light is relatively weak. The light-blocking rate of the KG film increases to 24.03% (at 700 nm). With the increase in HS content, the light-blocking rate of KGH_3_ reaches the optimal value: 56.70% at 400 nm and still remains at 49.70% at 700 nm, which is 162% higher than KGM. Due to the presence of aromatic amino acids in Gel with conjugated double bonds, it can efficiently absorb ultraviolet light (200–400 nm), which is the basis for the higher light-blocking rate of the KG film compared to KGM. The higher the amount of HS added, the stronger the absorption of visible light by -OH. Overall, adding Gel and HS can significantly enhance the light-blocking performance of the KGM-based films. Among them, KGH_3_ has the optimal barrier capabilities against ultraviolet and visible light.

Water vapor barrier property and oxygen barrier property are key indicators for food packaging. Notably, WVP is closely related to film thickness—thinner films typically exhibit higher WVP due to shorter water vapor diffusion paths. The thicknesses of KGM, KG, KGH_1_, KGH_2_, and KGH_3_ films are 105.29 μm, 81.22 μm, 68.07 μm, 55.09 μm, and 37.3 μm, respectively (Figure 6b). WVP values (Table 2) show KGM film had the lowest WVP (7.37 g·mm/m^2^·24 h·kPa), while Gel and HS addition significantly increased water vapor transmission rate. Thinner film thickness (from 105.29 μm to 37.3 μm) shortens the diffusion distance of water vapor molecules, further elevating WVP. This might be because the -O-CH_2_-CH(OH)-CH_3_ groups in HS molecules are hydrophilic; the introduction of HS brought in additional hydrophilic groups, further increasing the hydrophilic sites of the films. XRD and SEM (Figure 3 and Figure 4) confirmed that the increased HS content induced molecular chain bridging, resulting in a denser membrane interface. This enhanced oxygen diffusion resistance consequently improved the oxygen barrier properties of the membrane. OP values (Table 2) show that increasing HS content significantly decreased KG-based film OP from 8.73 to 3.14 g/m^2^·24 h, substantially reducing OP. XRD and SEM results (Figure 3 and Figure 4) verified that increased HS content created a molecular bridge effect among molecular chains, densifying the film interface. This increased resistance to oxygen diffusion, improving film oxygen barrier performance.

As shown in Table 2, compared with biobased materials (e.g., PLA, PHA) and petroleum-based LDPE in the table, KGH_3_’s WVP (12.23 g·mm/m^2^·24 h·kPa) is lower than brittle PLA (17.3 g·mm/m^2^·24 h·kPa), while its OP (3.14 g/m^2^·24 h) is close to PHA and superior to PLA; though LDPE has better barrier properties, KGH_3_’s biodegradability and rapid solubility make it a more sustainable alternative for targeted food packaging.

**Table 2:** Comparison of WVP and OP between KGH films and commercial plastics.

Material	WVP (g·mm/m^2^·24 h·kPa)	OP (g/m^2^·24 h)	Reference
KGM	7.27	8.48	-
KG	9.30	6.41	-
KGH_1_	10.61	5.43	-
KGH_2_	10.80	4.01	-
KGH_3_	12.23	3.14	-
PLA	17.3	4.1	[58]
PHA	4.7	1.5	[59]
PBSA	3.6	1.3	[60]
LDPE	2.0	0.007	[61]

### 3.5. Antioxidant Property of Films

**Figure 8:**
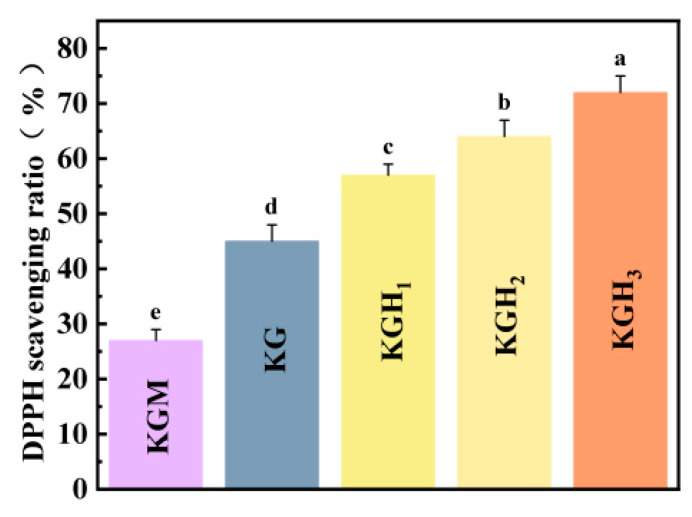
DPPH radical scavenging rates of different films. Different lowercase letters (a–e) indicate significant differences (*p* < 0.05) among groups.

Figure 8 shows that the addition of Gel and HS significantly increased the scavenging rate of DPPH free radicals by the membrane. As the amount of HS added increased, the scavenging rate of DPPH free radicals showed a significant upward trend. The scavenging rate of the pure KGM membrane was only about 27%, demonstrating the basic antioxidant capacity [62]. After introducing Gel, the scavenging rate of the KG membrane increased to approximately 45%; as the content of HS increased, the scavenging rates of KGH_1_, KGH_2_, and KGH_3_ successively rose to 57%, 64%, and 72%, among which the scavenging rate of KGH_3_ reached the highest (72%). From the FTIR results (Figure 1a), it can be seen that the -O-CH_2_-CH (OH)-CH_3_ of HS forms hydrogen bonds with the -OH of KGM and the -NH_2_ of Gel, destroying the originally tight intermolecular hydrogen bond network of KGM, thereby exposing more free -OH/-NH_2_ active sites, becoming the proton donor for DPPH free radicals, and thus determining the antioxidant capacity [63]. The aromatic amino acids in Gel (such as tyrosine and tryptophan) contain conjugated double bond structures, and the -OH groups in their side chains are themselves efficient radical scavenging sites. For example, the scavenging rate of the KG membrane increased to 45%, which is the direct contribution of the Gel active sites. From the XRD results (Figure 3), it is shown that the addition of HS reduced the crystallinity of KGM, transforming from an ordered crystal to an amorphous structure. The molecular chains in the amorphous region were arranged more loosely, further enhancing the accessibility of the active sites. This indicates that the effect of Gel and HS can significantly enhance the antioxidant performance of the membrane, and the addition amount of HS is positively correlated with the scavenging rate.

### 3.6. Film Seasoning Oil Packaging Application

**Figure 9:**
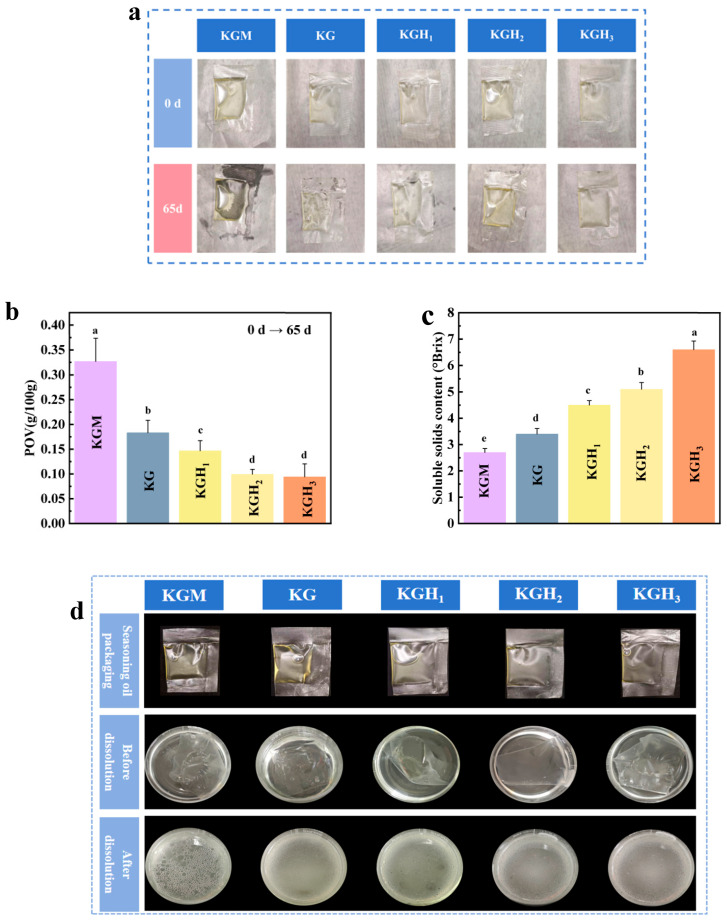
(**a**) Comparison of edible oil packages with different films before and after storage; (**b**) POV of edible oil packaged with different films; (**c**) SSC of edible oil packaged with different films; (**d**) comparison of the dissolution of oil encapsulated in different membranes in 100 °C distilled water. Different lowercase letters (a–e) indicate significant differences (*p* < 0.05) among groups.

Peanut oil is prone to oxidative rancidity during storage, which degrades sensory quality, reduces nutritional value, and generates harmful substances. Figure 9a compares different films after 65 days of storage. The poor heat-sealing effect of pure KGM film is evident, while HS addition enhanced heat sealing, consistent with FTIR and SEM results (Figure 1a and Figure 4).

Figure 9b shows POVs of 5 mL edible oil packaged in different films after storage at 25 °C in the dark for 65 days (initial POV ≤ 0.01 g/100 g). POV data show that after 65 days of dark storage, pure KGM film-packaged oil deteriorated (POV ≥ 0.25 g/100 g), while KGH film-packaged oil remained stable. This result might be because the pure KGM film had the lowest heat-sealing ability (Figure 5a) and OP value (Table 2), which increased the contact area with oxygen. As shown in Figure 9b, compared with KG, the POVs of peanut oil packaged with KGH_1_, KGH_2_, and KGH_3_ decreased by 19.9%, 45.8%, and 48.7%, respectively. This protection originates from a triple effect: (i) enhanced heat sealing minimized oxygen ingress [64], (ii) reduced OP limited diffusion, and (iii) intrinsic antioxidant groups scavenged free radicals. The 65-day shelf-life extension achieved without synthetic additives underscores KGH films’ potential for high-fat food preservation.

Figure 9c shows the data of SSC after the oil-in-water emulsions were dissolved by different membranes. The SSC of the pure KGM membrane reached 2.7 °Brix. After the introduction of Gel, the SSC increased to 3.4 °Brix. Although the hydrogen bond interaction between Gel and KGM strengthened the intermolecular binding, it also made some hydrogen bonds more prone to dissociation in hot water, promoting the release of a small amount of soluble components. As the content of HS increased, the SSC continued to rise, and finally reached 6.6 °Brix in KGH_3_ (an increase of approximately 94.1% compared to KG). Due to the -CH_2_CH(OH)CH_3_ side chain of HS breaking the ordered aggregation structure of the molecular chain and reducing the intermolecular forces, the film became more soluble in hot water, and the release amount of soluble solids (such as polysaccharide fragments, small molecule additives) significantly increased with the increase in HS addition. This trend was completely consistent with the results of FTIR (Figure 1a) (HS breaks hydrogen bonds and widens the peak width). With the increase in HS content, the final result was a continuous increase in the SSC value, with KGH_3_ having the best dissolution performance.

## 4. Conclusions

This study successfully engineered KGM-based films with adhesive-free heat-sealing capability and rapid dissolution properties through strategic incorporation of Gel and HS. Comprehensive characterization elucidated a clear structure–property paradigm driven by the thermoplastic effect of HS. Specifically, HS’s hydroxypropyl side chains exert dual functions: (1) they disrupt the rigid, ordered hydrogen bond network of KGM and Gel, acting as “internal molecular plasticizers” to enhance molecular chain mobility; (2) they serve as bridging agents to form a flexible three-dimensional hydrogen-bonded network between KGM, Gel, and HS molecules. This thermoplastic regulation not only improves interfacial compatibility and fusion (enabling adhesive-free heat-sealing) but also loosens the film’s microstructure (facilitating rapid dissolution), while synchronously enhancing mechanical strength. The optimized KGH_3_ formulation achieves a 93% improvement in heat-seal strength, 70% faster dissolution, and 48.7% reduction in oil oxidation over 65 days compared to conventional KG films. Compared to fossil-based packaging materials, these films offer inherent advantages in renewability, potential biodegradability, and food safety, addressing critical environmental and practical concerns in the convenience food packaging sector.

Notably, while the films’ components are inherently biodegradable and the literature supports potential compliance with the Test scheme and evaluation criteria for the final acceptance of packaging [65], formal biodegradability testing against this threshold was not conducted herein. Future work will prioritize the following: (1) EN 13432-compliant biodegradation and disintegration testing to validate environmental sustainability; (2) enhancing long-term humidity stability; (3) scaling production to translate laboratory advances into industrial applications. This research provides a mechanistic foundation for the rational design of next-generation biodegradable packaging materials that reconcile performance demands with sustainability imperatives.

## Data Availability

The raw data supporting the conclusions of this article will be made available by the authors on request.
